# An investigation into the anthropogenic nexus among consumption of energy, tourism, and economic growth: do economic policy uncertainties matter?

**DOI:** 10.1007/s11356-020-10638-x

**Published:** 2020-09-07

**Authors:** Festus Fatai Adedoyin, Solomon Nathaniel, Ngozi Adeleye

**Affiliations:** 1 Department of Accounting, Finance and Economics, Bournemouth University, Poole, UK; 2 Department of Economics, University of Lagos, Lagos, Nigeria; 3 School of Foundation, Lagos State University, Badagry, Nigeria; 4 Department of Economics and Development Studies, Covenant University, Ota, Nigeria; 5 Regional Centre of Expertise (RCE), Ota, Ogun Nigeria

**Keywords:** Economic policy uncertainties, Tourist arrivals, Energy use, Ecological footprints, Economic growth

## Abstract

Global warming has been a pressing issue for the past decade as various economic activities have been flagged and are expected to reduce emissions. While previous studies have examined the energy consumption-emissions-economic growth nexus in significant detail, attention is yet to be given to the role of economic policy uncertainties and human activities such as tourism in a carbon function. Thus, this study aims to investigate the long-run relationship between energy consumption, tourists’ arrivals, economic policy uncertainty, and ecological footprint in the top ten earners from international tourism over the period 1995 to 2015. The fully modified ordinary least square and dynamic ordinary least square estimation techniques and the Dumitrescu and Hurlin causality tests were used in the study. Empirical results suggest that economic policy uncertainties in addition to tourism and energy consumption are drivers of environmental degradation. However, the contribution of energy consumption to ecological footprint is significantly moderated by economic policy uncertainties such that a 1% increase in the latter reduces environmental damage by 0.71%. This study suggests that policy uncertainties matter a great deal for energy and environmental policies. Also, green economic growth is possible if the proper implementation of environmental protection policies can restrict the harmful impact of economic activities on the quality of the environment. Based on the empirical findings, vital energy policy recommendations are suggested.

## Introduction

The availability of energy resources is generally perceived to be one of the major drivers of economic growth and development in many industrialised economies. This draws from the evidence that energy consumption actively contributes to economic growth (Adedoyin et al. 2020a, b; Kirikkaleli et al. 2020; Udi et al. 2020; Nathaniel et al. 2020c); hence, countries which are impoverished in terms of energy resources are potentially faced with the syndrome of negative economic growth. However, the continuous exploitation of energy resources by man is putting the ecological environment on terrific pressure. Consequently, there have been several cases of ecological impediments such as environmental pollution, ecological degradation, and global warming, and a host of other complications that serve as threats to the survival of humans as well as economic growth and development of the global economy (Nathaniel and Khan 2020). In this regard, pollution from non-renewable sources such as fossils, fuels, coal, and firewood and a host of others have caused terminal illnesses as well as death to humans (Guarnieri and Balmes 2014). In recent years, many economic literature have focused on the impact of energy resources on economic growth and development to the causal connection of the two aforementioned variables to environmental sustainability, and this hot debate is tantamount to what is generally known as environmental Kuznets curve (EKC) hypothesis (Dogan et al. 2020; Dogan and Inglesi-Lotz 2020).

The EKC hypothesis is an inverted U-shaped graphical representation that depicts how the continuous search for economic growth will conform to environmental degradation at the early stages of development until it reaches a saturated point where the nexus between economic growth and environmental pollution becomes negative (Işık et al. 2019a, b). A study conducted by Bölük and Mert (2015) encapsulates the U-shaped curve as a nexus that occurs when a country reaches a certain level of development where there is abundant availability of growth-enhancing technologies that can be channelled towards increasing the efficiency of energy resources in lieu of renewable energy. Moreover, the focus of energy policy is to upsurge the efficiency of renewable energy such as wind, solar, biomass, and geothermal, to replace the non-renewable energy which is generally considered as the reason behind the environmental degradation (Isik et al. 2018).

The increased exploitation of energy resources by man is now resulting in the increasing absorptions of the CO_2_ by the world climate (Adedoyin et al. 2020c, d, e; Etokakpan et al. 2020; Nathaniel et al. 2020d). Many countries in the world are experiencing significant changes in climatic conditions due to overdependence on a fossil energy source which allows for the increasing greenhouse emission (Işik et al. 2017b; Pandey et al. 2020). Thus, many policymakers and researchers are working earnestly to address the issues of energy resource consumption and CO_2_ emission which are putting the global climate at risk. In recent time, the tourism sector has become a global economic sector that is in line with many other economic sectors in the world such as accommodation, aviation, and trade, and it has contributed a lot to the significant climate change experienced in the world (Dogru et al. 2019). The theoretical support for the tourism-growth-energy-environment nexus rests on the EKC. The EKC has become popular since the seminal work of Kraft and Kraft (1978) on the link between energy use and GNP using the USA as a case study. In recent times, tourism demand via international tourist/tourist receipt has been recognised as a driver of pollutant emissions and consequently worsening the quality of the environment (Işik et al. 2017a, b, 2019a; Gokmenoglu and Eren 2020).

In the same vein, the debate of the causal relationship between CO_2_ emission and tourism sector led to the concept of tourist carbon footprint (TCF). The TCF is an index for measuring the personal consumption, transportation, activities, and accommodation cost of tourists. The economic benefits of tourism sector cannot be overemphasised as the sector contributes significantly to the global gross domestic product. International arrivals and tourism receipts have been experiencing rapid growth at an annual growth rate of 3.3% and a contribution of 1.3 trillion dollars to global exports (UNTWO 2015). Unarguably, an economic sector at this level of productivity has a role to play on the environment. Specifically, transport as an important ingredient of the tourism sector is an energy-consuming and carbon-intensive product, tagging the tourism sector as a potential contributor to world climate change. The susceptibility of tourists’ destinations to climate change is an indication that there is a likelihood of the tourism industry undergoing significant changes. Akin to this, tourism carbon footprints include the greenhouse gasses (GHGs) emitted in the course of tourism activities such as the carbon embedded in tourism-related goods and services.

However, the pushing factors of tourism-energy-emission nexus and environmental sustainability are being debated vigorously in the literature. One of which is economic policy uncertainties (EPU). The EPU index is used to measure the economic risk attached to the future path of unreliable government policy which delays the decision of investors to invest until the uncertainty is resolved. Given the recent global financial crisis and the growing concerns for policy disputes, it is important for policymakers to focus on the channels through which EPU have rotten the relationship between tourism-energy-emission nexus and environmental sustainability. The feedback effect of the policy uncertainties comprises issues such as unemployment and income inequality crisis, migration crisis, and other challenges that are furthering the complications of the global economies. Moreover, it has become more evident from the recent downturn of economic growth in many countries that policy uncertainties are highly significant in shaping economic aftermaths.

Given the foregoing, this study contributes to the literature firstly by introducing economic policy uncertainties in the energy consumption-emission nexus, and secondly by capturing this causal linkage in the context of higher human activities in the form of tourism consumption. Put succinctly, this study analyses causal linkage among energy consumption, tourists’ arrivals, economic expansion, and environmental sustainability with a specific focus on the impacts of economic policy uncertainties on the ecological footprint in countries with the highest revenue from the tourism industry. As such, the study gives room for a better understanding on how other factors such as policy uncertainty plays in the tourism-growth-energy-environment nexus. Such an investigation is necessary given the fact that the role of economic policy uncertainties in the tourism-growth-energy-environment nexus has been scarcely investigated empirically (Işık et al. 2019b). Furthermore, the study also aims to come up with effective energy policies that can be employed to address the issues evolving around carbon emission and environment degradation arising from the tourism industry of the sample countries examined in the study and the global economy at large. In line with the ongoing discussion, the following research questions are posed:

Research question 1:

Is there a long-run relationship between tourism, economic policy uncertainty, growth, energy consumption, and the environment?

Research question 2:

Is there causality between the variables under review?

The “Literature review and stylized facts” section presents a detailed review of the literature on the connection between tourism and economic policy uncertainties; tourism and energy consumption; tourism and economic growth; and tourism and environmental degradation. This is followed by the “Data and methods” section which presents data and methodology used to achieve the objective of the study. The “Results and discussions” section discusses the results of the study with the economic and environmental implications of research findings. The study ends with the “Conclusion” section with vital policy recommendations.

## Literature review and stylized facts

According to World Tourism Organization, Annual (2016) Report, most tourists are attracted to Europe which represents half of the world’s international tourists’ destinations and the top 5 visited countries are France, Spain, Italy, Turkey, Germany, and the UK. Since the empirical findings of the tourism sector are fast gaining relevance, the review of the literature is structured in two parts. The first part without claiming to be exhaustive discusses the direction of causality in the tourism-growth and tourism-energy nexus. According to Antonakakis et al. (2016) and Nepal et al. (2019), the tourism-growth relation can be examined from four perspectives: (1) does tourism lead growth?, (2) is tourism economy-driven?, (3) are there feedbacks?, and (4) no causal relationship exists. Thereafter, an expose of the tourism-economic policy uncertainty discourse is engaged.

### Tourism and economic growth

Depending on the scope of the study, variables used, and analytical techniques adopted, empirical findings are mixed regarding the nature of causal relationships between tourism and economic growth (Pablo-Romero and Molina 2013). The absence of a clear agreement on the precise nature of causality indicates that the research area requires more empirical investigations. On a panel of 32 non-OECD countries, Lee and Chang (2008) find a bidirectional causal link between tourism and growth. Isik and Radulescu (2017) find a bidirectional causal relationship between tourist arrivals and growth for Greece. Supportively, Chen and Chiou-Wei (2009) find similar evidence that bidirectional causal relationship exists in a study of Taiwan and Korea. Other studies that support the bidirectional causal relationships (Cortes-Jimenez and Pulina 2010; Seetanah 2011; Nissan et al. 2011; Caglayan et al. 2012; Aslan 2015). Likewise, Paudyal (2012) finds a bidirectional relationship between tourism receipts and output in Nepal.

Similar findings from Gautam (2011) on a long- and short-run bi-causal relationship between tourism receipts and GDP in Nepal. Findings on the unidirectional causality are also mixed between growth-led-tourism (GLT) and tourism-led-growth (TLG) relations. Using a bivariate model of tourism receipts and output from South Korea, Oh (2005) provides evidence that growth leads to tourism. From the study on Cyprus, Katircioglu (2009) finds evidence that supports the growth-led-tourism hypothesis from using the autoregressive distributed lag (ARDL) model technique and the Granger test for causality; the study shows that economic growth Granger-causes tourism. Equally, by deploying the Toda-Yamamoto causality test on a study of the tourism sector and economic growth in Croatia, Mervar (2010) finds evidence to support the argument that growth leads to tourism. In the same vein, Tang (2011), Massidda and Mattana (2012), and Lean and Tang (2012) corroborate the growth-led-tourism hypothesis for Malaysia; Cortés-Jiménez et al. (2011) for Tunisia; Kum et al. (2015) from a panel of 11 countries; and Lanza et al. (2003) from 13 OECD countries. Several studies also support the argument that tourism leads growth for Pakistan (Adnan Hye and Ali Khan 2013; Khalil et al. 2007) and Sri Lanka (Srinivasan et al. 2012). Bilen et al. (2015) using the Dumitrescu-Hurlin causality test also show that the tourism-led-hypothesis holds from a study on 12 Mediterranean countries.

### Energy consumption and tourism

Relative to the tourism-growth studies, those specific and direct on the tourism-energy relation are sparse and developing. However, the consensus is that tourism and energy consumption have a synergetic relationship with the tourism industry being fuel-dependent and a contributor to greenhouse gas (GHG) emissions (Gössling 2013). On average, a tourist is expected to make use of some medium of international travels, local transportation, accommodation, and feeding (Gössling et al. 2015; Gössling and Peeters 2015). These activities involve different forms of energy use from renewable, non-renewable, fossil fuels, charcoals, and wood (Becken and Simmons 2005). Hence, it is expected that tourism attracts huge quantities of energy usage.

We summarise a few of the tourism-energy studies. On a study of Malaysia, Solarin (2014) showed that a unidirectional long-run causality exists between tourist arrivals and energy usage. Using a trivariate model, Katircioglu (2014) showed that in the long run, increased energy usage is associated with an increased number of tourist arrivals in Turkey. Also, on a similar study on Cyprus, Katircioglu et al. (2014) reveal that in the long run, international tourist arrivals have a positive inelastic relationship with the level of energy consumption. Likewise, from a trivariate analysis, Tang et al. (2016) showed energy consumption is driven by tourism in the long run in India. Işik et al. (2017b) find a bidirectional causal relationship between tourist arrivals and energy consumption in ten most visited countries in the world. Likewise, in rural Nepal, findings reveal that tourists’ activities increased the consumption of primary energy sources like wood and kerosene (Nepal 2008). In fact, tourist destinations amass considerable quantities of energy to import supplies, transport water, and dispose wastes. This assertion as supported by Dwyer et al. (2010) evidence that tourists’ resorts and parks are the areas of high-energy usage due to the use of automated activities. Although tourism is closely related to environmental activities, only a few studies highlight the possible effect of tourism on the environment, more so with mixed findings (De Vita et al. 2015; Dogan et al. 2015).

### Environmental degradation and tourism

On the contributions of tourism to the environment, Gössling (2000) documents that tourism-related use of fossil fuels has adverse environmental consequences. Likewise, Gössling et al. (2015) show that of recent, an increase in air travel has somewhat increased the contribution of the aviation sector to increasing global CO_2_ emissions. Koçak et al. (2020) provide evidence that tourism arrivals have an increasing effect on CO_2_ emissions while tourism receipts have a reducing effect on CO_2_ emissions and that a possible co-movement and causal relationship exist between tourist activities and CO_2_ emissions in the long run. Another channel by which tourism contributes to environmental degradation is through changes in land-use as a result of tourism investments. That is, land-use change results in tree-felling activities and decrease in forest areas which coincidentally contributes to CO_2_ emissions and fossil energy (Al-Mulali et al. 2015; Raza et al. 2017; Sharif et al. 2017; Zaman et al. 2017; Bilgili et al. 2017; Pandey et al. 2020). On the other hand, the literature documents studies that posit a well-managed tourism sector can promote an eco-friendly environment through the usage of friendly technology and transportation (Paramati et al. 2017a). They argue that wider and safer roads coupled with rail and sea transportation will reduce CO_2_ emissions and ensure environmental quality (Paramati et al. 2018).

### Tourism and economic policy uncertainties

The tourism sector is susceptible to international shocks, crises, and disasters such as the London and Bali bombings, Asia’s bird flu, America’s Hurricane Katrina, US-China Trade Wars, and of recent the coronavirus pandemic from Wuhan, China. These crises fuel uncertainties either from home or destination countries and create the unwillingness of tourists to travel and spend (Maki 1998). In other words, uncertainties impede tourism. The measure of uncertainty is inconsistent in the literature as several indicators have been used such as volatility index (VIX), stock market volatility, geopolitical risks, economic growth, and political risks. Of recent, Baker et al. (2016), using different components, formulated a relatively new economic policy uncertainty (EPU) index from several snapshots of economic policy uncertainty over time (Ongan and Gozgor 2017). The EPU captures the uncertainty from the policymakers and those affected by the economic effect of those policies (Akron et al. 2020). A growing literature on the impact of uncertainties on the tourism sector documents the evidence using economic, financial, health, and climatic factors. For instance, on epidemic disease-related and natural disaster uncertainties (see, e.g., Chen et al. 2017; Chew and Jahari 2014; Morakabati and Kapuściński 2016; Wang 2009), and uncertainties from terrorism and crime-related human factors (see Karl et al. 2017; Saha and Yap 2014) Much of these studies model the impacts of these crises on the tourism sector using dummy variables (Ongan and Gozgor 2017).

However, the EPU index successfully incorporates all economic, financial, health, and climatic factors which give it an edge as an all-encompassing variable over the qualitative crisis and policy change indicators. We examine the literature on the relation between the EPU and the tourism or hospitality industry. The EPU in the USA has a negative impact on US outbound tourists (Dragouni et al. 2016). Likewise, the EPU in the USA is negatively associated with domestic tourism spending in the USA (Gozgor and Ongan 2017). According to Işık et al. (2019c), an increase in the EPU in Canada and Mexico reduces the number of tourists’ arrivals from these countries to the USA. Similarly, Akron et al. 2020) find that investment policies in 305 hospitality companies are negatively affected by EPU in the USA. Hospitality firms are conventionally capital-intensive and increasing economic uncertainties is likely to drive down, postpone, or cancel the provision of physical infrastructures which will directly impact tourists’ arrivals. This is because the decline or non-existent infrastructures will hamper the abilities of hospitality firms to satisfactorily meet tourists’ demand creating less-satisfied customers and a reduction in tourists’ receipts (Turner and Hesford 2019). In other words, in the event of uncertainties, the option value of waiting for better information increases (Dixit and Pindyck 1994). Empirical evidence that the tourism sector is sensitive to EPU has been documented by Demir and Ersan (2018), Demir and Gozgor (2018), Dragouni et al. (2016), Ersan et al. (2019), Madanoglu and Ozdemir (2019), and Wu and Wu (2019) to mention a few.

## Data and methods

### Data and variables

The data for this study covers the top ten earners from tourism based on their tourism revenue over the period 1995 to 2015. This is due to the limitation in the availability of data for the selected variables, i.e. ecological footprint in particular. These countries of focus according to their 2017 international tourism, receipts (US$’ million) include the USA (251,361); the UK (51,474); France (69,894); Italy (44,548); Spain (68,437); Australia (43,982); Thailand (62,158); Hong Kong SAR, China (38,039); Germany (56,173); and Japan (36,979).^1^ The choice of 2017 data for tourism is due to the latest available data for tourism revenue which motivates our choice of countries for the study. Data is also collected for the following variables:^2^ total ecological footprint measured in area per capita sourced from the Global Footprint Network (2019); real gross domestic product per capita measured in constant 2010 US$; international tourist arrivals (numbers); energy use; and economic policy uncertainty which is proxied by the world uncertainty index and is sourced from Ahir et al. (2018) (http://www.policyuncertainty.com).

### Stylized facts

This part of the study outlines some stylized facts on the 10 countries and variables used in this study. This involves drawing out similarities and differences in their patterns of behaviour concerning tourist arrivals, energy consumption, economic policy uncertainties, and ecological footprint.

**Fig. 1:**
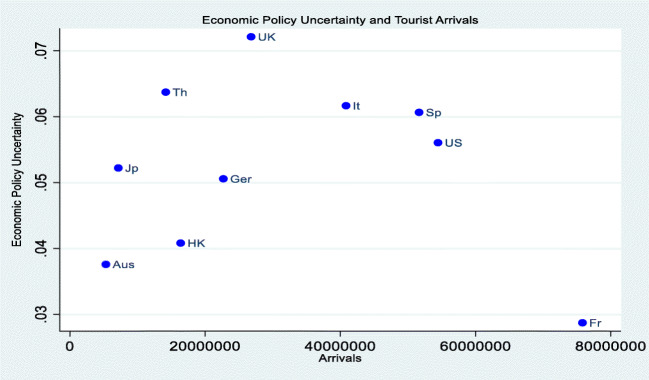
Economic policy uncertainty and tourism arrival relation. Source: authors

From the available data, although there seems to be no obvious pattern on the relationship between economic policy uncertainty (EPU) index and average number of tourist arrivals, it can be drawn that low uncertainties drive the tourism sector. From Fig. 1, the UK has the highest EPU index of 0.072 with a corresponding average number of tourists’ arrivals at 26.79 million relative to France with the lowest EPU index of 0.028 and a number of tourists’ arrivals at 75.8 million. Therefore, the supposition that uncertainty is negatively associated with tourism is evident in the data, though subject to econometric testing.

**Fig. 2:**
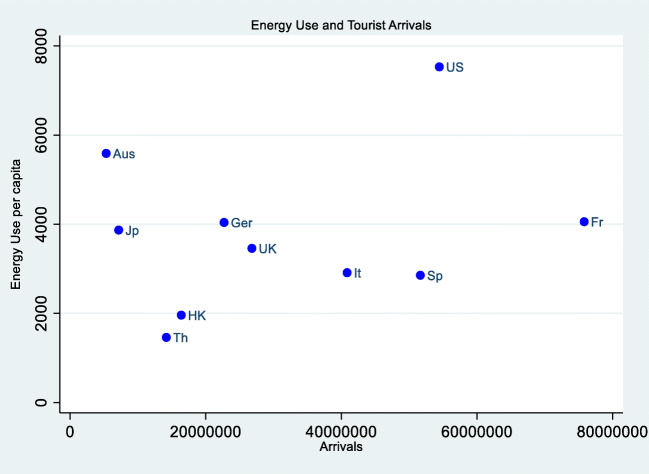
Energy use and tourism arrival relation. Source: authors

Empirical evidence supports the argument that the tourism sector uses all forms of energy needs ranging from oil, natural gas, coal, to fossil fuel to sustain its involvement in transportation, accommodation, and general logistics (Nepal et al. 2019; Gössling and Peeters 2015). In Fig. 2, we show that countries with high tourist arrivals consume high energy. For instance, the USA, on the average, has 7530.89 energy use per capita and a corresponding average tourists’ arrival of 54.4 million. However, we do not find that countries with low energy consumption have low number of tourists’ arrivals. Thailand has the lowest average energy use per capita at 1460.72 with 14.18 million average tourists’ arrivals while Australia has the second highest average energy use per capita at 5588.26 but has the lowest number of tourists’ arrivals at 5.32 million.

**Fig. 3:**
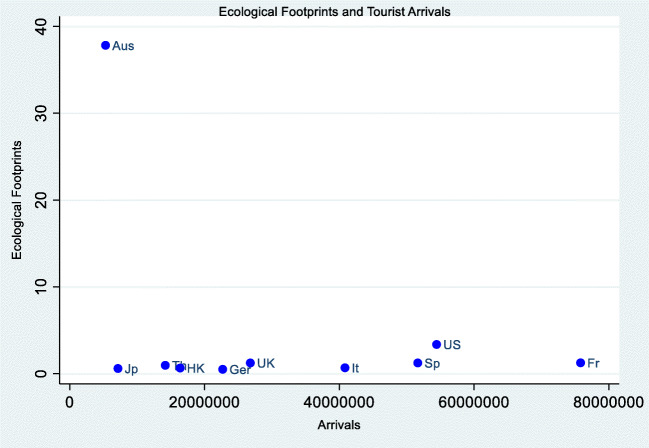
Ecological footprints and tourism arrival relation. Source: authors

It is expected that increased energy usage is in tandem with tourism activities (Dogan and Aslan 2017). As tourism involves the usage of different means of transformation encompassing the consumption of different types of energy may either increase or decrease the environmental pollution. We draw out the ecological footprints (EFP) and tourists’ arrival relation. Figure 3 shows that all countries, with the exception of Australia, have low EFP of between 0.5 and 3.36.

### Summary statistics and correlation analysis

Table 1 shows the summary statistics (upper panel) using the raw variables and the pairwise correlation analysis (lower panel) using the log-transformation of the variables. The justification for using the raw forms of the variables and not the log-transformation to conduct the descriptive statistics is because all historical information about the variable will be lost and inaccurate information obtained about the measures of central tendency, measures of dispersion etc. Similarly, since the log-log functional form of the model is estimated it is justifiable to observe the associations of the variables in their natural logarithms to ensure that the problem of multicollinearity is averted.

**Table 1:** Summary statistics and correlation matrix

Variables	EFP	EPU	GDP	TOA	EU
Mean	4.83	0.05	35,201.18	31,745,570.05	3792.65
Standard deviation	11.15	0.03	12,508.32	23,157,421.31	1690.18
Minimum	0.49	0.00	3236.37	3,345,000.00	1041.31
Maximum	44.51	0.15	55,079.89	84,452,000.00	8056.86
Correlation matrix					
Ecological footprints (log)	1.000				
Economic policy uncertainty (log)	− 0.134*	1.000			
Per capita GDP (log)	0.227***	− 0.101	1.000		
Tourist arrivals (log)	− 0.296***	0.046	0.164**	1.000	
Energy use per capita (log)	0.518***	− 0.156**	0.776***	0.118*	1.000

***, **, and * represent statistical significance at the 1%, 5%, and 10%, respectively

*EFP* ecological footprint, *EPU* economic policy uncertainty, *GDP* per capita GDP, *TOA* tourists’ arrivals, *EU* energy use per capita. Source: authors’ computations

The average value of ecological footprint is 4.83, which is between 0.49 and 44.51 with a standard deviation of 11.15 which suggests the countries show wide dispersion from the sample average. The mean value of economic policy uncertainty is 0.05 with a variability of 0.03 and ranges between 0.00 and 0.15. Similarly, per capita GDP has a mean score of US$35,201.18 with minimum and maximum values of US$3236.37 and US$55,079.89 and a variability of 23,157,421.31 indicating a huge deviation from the sample mean. Tourists’ arrivals have a mean value of 31,745,570.05; has a variability of 2,315,7421.31; and ranges between 3,345,000.00 and 8,4452,000.00. On average, energy use per capita has a mean score of 3792.65 and a variability of 1690.18.

From the lower panel of Table 1, the correlation matrix shows that all the explanatory variables have statistically significant associations of 1% and 10% levels with the dependent variable, ecological footprint. While economic policy uncertainty and tourists’ arrivals are negatively associated; per capita GDP and energy use per capita are positively associated with ecological footprint. Notably, energy use per capita shows the highest coefficient of correlation, which highlights that energy use per capita has a considerable effect on ecological footprint among the top destination countries.

### Model and methods

A number of studies in the literature have investigated the tourism-emission nexus (Raza et al. 2017; Sharif et al. 2017; Zaman et al. 2017; Bilgili et al. 2017). While several others have been conducted on tourism-energy use nexus (De Vita et al. 2015; Dogan et al. 2015), while yet some have focused on tourism-economic growth link (Cortes-Jimenez and Pulina 2010; Seetanah 2011; Nissan et al. 2011; Caglayan et al. 2012; Aslan 2015) and some on tourism and economic policy relationship (Chen et al. 2017; Chew and Jahari 2014; Morakabati and Kapuściński 2016; Wang 2009; Karl et al. 2017; Saha and Yap 2014; Ongan and Gozgor 2017)

However, this study differs by introducing economic policy uncertainty as a variable into a model with tourism, economic growth, and energy use as determinants of environmental quality. Also important is that our study uses ecological footprints instead of CO_2_ so as to present some newness different from other studies on tourism-energy-emission nexus. In addition, the study investigates the EKC hypothesis in the ten countries such that the model equations are as follows:

1 $$ \mathrm{EFP}=f\left(\mathrm{GDP},\mathrm{GDP}2,\mathrm{TOA},\mathrm{EU}\right) $$

2 $$ \mathrm{EFP}=f\left(\mathrm{GDP},\mathrm{GDP}2,\mathrm{TOA},\mathrm{EU},\mathrm{EPU},\mathrm{EU}\ast \mathrm{EPU}\right) $$

3 $$ \mathrm{LEFP}={\alpha}_0+{\beta}_1{\mathrm{GDP}}_{it}+{\beta}_2{\mathrm{GDP}\mathrm{sq}}_{it}+{\beta}_3{\mathrm{TOA}}_{it}+{\beta}_4{\mathrm{EU}}_{it}+{\beta}_5{\mathrm{EPU}}_{it}+{\beta}_6{\mathrm{EU}\mathrm{EPU}}_{it}+{\varepsilon}_{it} $$

The study adopts a logarithmic transformation of all variables to ensure that the variance remains constant across all the series. Where EFP (log), GDP (log), GDPsq (log), EU (log), EPU (log), and TOA (log) are logarithmic transformations of all variables and *ε*_*it*_ , α, and *β*s represent the stochastic, intercept, and partial slope coefficients, respectively.

We adopt the fully modified ordinary least square (FMOLS) and dynamic ordinary least square (DOLS), by Pedroni (2001) and Kao and Chiang (2000), respectively, for estimation in this study. This is because the FMOLS and DOLS are very efficient for the estimation of a cointegrating panel. Fortunately, the DOLS while correcting for correlation between the dependent variable and the stochastic term, it also adds lags of the independent variables.

## Results and discussions

### Pre-estimation diagnostics

In Table 2, there is evidence of cross-sectional dependence. This informs the adoption of a second-generation unit root test (Table 3). The two unit root tests (CIPS and CADF) confirmed that the variables are *I*(1). With this result, the study proceeds with the Westerlund (2007) cointegration test which accounts for cross-sectional dependence (see Table 4). The results in Table 4 suggest the existence of a long-run relationship among the variables.

**Table 2:** Cross-sectional dependence test

Variables	Breusch-Pagan LM	Pesaran scaled LM	Pesaran CD
Ecological footprint (log)	756.4751***	74.99606***	27.30638***
GDP (log)	633.5613***	62.03981***	23.87695***
GDP squared (log)	272.5359***	23.98439***	8.838398***
EPU (log)	99.91598***	5.788652***	5.533197***
Energy use (log)	669.2875***	65.80568***	17.98990***
TOA (log)	733.0055***	72.27215***	27.00221***

*** implies statistical significance at the 1% level

**Table 3:** Panel unit root tests

Variables	Level		First difference	
	CIPS	CADF	CIPS	CADF
EFP (log)	− 2.231	23.44	− 3.352***	112.1***
GDP (log)	− 3.435	22.54	− 5.546***	92.31***
GDPsq. (log)	− 1.432	34.22	− 2.765***	76.54***
EU (log)	− 2.498	52.11	− 4.543***	59.12***
EPU (log)	− 3.845	34.67	− 5.653***	88.76***
TOA (log)	− 2.543	65.76	− 4.453***	88.45***

*** implies statistical significance at the 1% level. Source: authors’ computations

**Table 4:** Panel cointegration test (Westerlund)

Statistic	Value	Robust *P* value
Gt	− 1.371	0.995
Ga	− 15.917**	0.042
Pt	− 2.819	0.995
Pa	− 16.67*	0.077

* and ** show significance at 10% and 5% levels. Source: Author’s computation

### Estimation results

The results from the three tests are in harmony. They confirm that economic growth does not add to environmental degradation in these countries; rather, it improves environmental quality. Also, no evidence of the EKC hypothesis exists in the data. This revelation is intuitive as none of these countries is still at its initial stage of growth. These countries (Germany, Australia, France, Japan, Hong Kong, Italy, UK, Thailand, Spain, and the US) have gone past the stages) where growth can be detrimental to their economic advancement. Unlike economic growth, tourists’ arrivals are one of the drivers of environmental degradation in these countries. The arrival of tourist encourages an increase in tourism-induced activities which contribute to the degradation of the environment such as transportation services and construction of accommodation and other tourist facilities (Adedoyin and Bekun 2020). Also, most of these countries still harbor non-renewable (NRE) sources in their energy mix (Table 5).

**Table 5:** FMOLS, DOLS, and Driscoll/Kraay (dependent variable: EFP)

Variables	FMOLS	DOLS	Driscoll/Kraay
GDP (log)	− 0.3087***	− 0.8161***	− 2.2963***
	(− 8.0594)	(− 3.6424)	(− 8.26)
GDP squared (log)	0.0008	0.0882***	0.2760***
	(0.4450)	(3.2198)	(8.91)
Tourists’ arrivals (log)	0.0595***	0.2143**	0.3211***
	(5.0114)	(2.0248)	(14.77)
Energy use (log)	0.1535***	0.6225***	0.3052***
	(6.3069)	(3.2618)	(3.22)
Economic policy uncertainty (log)	0.0125***	0.0373**	0.3052
	(3.3845)	(2.1593)	(0.22)

*** and ** represent statistical significance at the 1% and 5% levels of significance, respectively. *t* statistics are in parentheses. Source: authors’ computations

Hence, the influx of tourists exacerbates the consumption of NRE which deteriorates the environment by increasing the ecological footprint. This finding complements those of Koçak et al. (2020), Dogan et al. (2017), Paramati et al. (2017), Akadiri et al. (2020), Shakouri et al. (2017), and Paramati et al. (2018) for ten most visited countries, OECD, Eastern and Western EU countries, 16 countries, Asia-Pacific countries, and in the EU countries, respectively. The influence of energy consumption (NRE) on the environment is similar to that of tourists’ arrivals but larger in magnitude. The findings suggest that NRE consumption is a major driver of environmental degradation in these countries. This is so because non-renewable energy comprises majorly of carbon-emitting energy resources which are harmful to the quality of the environment; hence, the increased use of these energy sources depletes the condition of the environment. This is actually in line with the findings of Nathaniel et al. (2020a) for CIVETS, Nathaniel et al. (2020b) for MENA, Destek and Sarkodie (2019) for N-11 countries, Dogan et al. (2019) for MINT, Ahmed et al. (2020) for G7 countries, and Nathaniel (2020) for Indonesia. Just like NRE, EPU also contributes to environmental degradation. This finding is intuitive and in line with our speculation that EPU can affect the environment through its impact on economic activities including investment, stock market, and trade, which aligns with the findings of Adedoyin and Zakari (2020). Similarly, times of policy uncertainty entails poor implementation of policies meant to safeguard the quality of the environment, hence the perpetuation of activities capable of harming the environment by economic agents. The horrendous influence of policy uncertainty on the environment has been confirmed by Bergen and Muñoz (2018) for Chile.

In Table 6, where we have tried to examine the moderating role of EPU on the environment, no evidence of the EKC was evident in the data. Going further, the influence of tourists’ arrival on the EFP is negative, which entails the possibility of the adoption of environmentally safe tourism policies such the use of green transport—for instance electric trains—by tourists. However, NRE and EPU still exact a detrimental impact on the environment by increasing the EFP. The result in Table 6 further revealed that the interaction effect between energy use and economic policy uncertainty (EU × EPU) has a negative coefficient. This outcome helps to support our hypothesis that energy use (NRE) is linked to EPU and the EFP. This suffices to say that when NRE is consumed, EPU achieves the level required to decrease the EFP. This effect points to the fact that the biologically productive land is not, in general, over-exploited.

**Table 6:** Moderating role of energy consumption on economic policy uncertainty (dependent variable: EFP)

Variables	FMOLS	DOLS
GDP (log)	0.0374	0.2558***
	(0.2292)	(4.3055)
GDP squared (log)	0.0536***	0.0273***
	(2.8126)	(3.7345)
Tourists’ arrivals (log)	− 0.3832***	− 0.3843***
	(− 5.2457)	(− 20.885)
Energy use (log)	0.8080***	0.5999***
	(3.659)	(7.5469)
Economic policy uncertainty (log)	1.3743***	1.4745**
	(4.6908)	(8.4573)
EU × EPU (log)	− 0.7061***	− 0.7561***
	(− 4.7078)	(− 9.1534)

*** and ** represent statistical significance at the 1% and 5% levels of significance, respectively. *t* statistics are in parentheses. Source: authors’ computations

### Dumitrescu and Hurlin causality tests

The evidence of cointegration among the variables in this study point to the presence of at least one causality link between the variables. To determine the direction of causality among the interest variables, we adopt the Dumitrescu and Hurlin causality tests (Table 7). With this test, we can determine which variable serves as a causative agent to the other. Results from the test reveal that no direction of causality was discovered between EFP and EPU, GDP and EPU, TOA and GDP, EU, and EPU. However, a feedback causality was discovered between GDP and EFP, and between EFP and the square of GDP. The same direction of causality exists between EU and the square of GDP. On the other hand, EU drives EPU, while GDP drives EU. A unidirectional causality also flows from TOA to GDP. The outcome here suggests that tourism development is responsible for the growth in these economies, and growth drives environmental deterioration. While comparing the causality results with previous findings, the feedback causality between EFP and GDP is similar to the findings of Bello et al. (2018) for Malaysia. On the other hand, the unidirectional causality from tourist arrivals to GDP is congruent to the findings of Katircioglu (2009) for Cyprus.

**Table 7:** Dumitrescu and Hurlin causality results

Null hypothesis	W-stat.	Zbar-stat.	Prob.	Conclusion
EPU ≠ > EFP	0.5685	− 1.0044	0.3152	No causality
EFP ≠ > EPU	3.7460	4.6466	3.E-06	
EU ≠ > EFP	4.9812	6.8433	8.E-12	Unidirectional causality
EFP ≠ > EU	3.1181	3.5298	0.0004	
GDP ≠ > EFP	7.2427	10.865	0.0000	Bidirectional causality
EFP ≠ > GDP	2.7708	2.9122	0.0036	
GDPsq ≠ > EFP	2.5982	2.6052	0.0092	Bidirectional causality
EFP ≠ > GDPsq	12.410	20.055	0.0000	
EU ≠ > EPU	2.8543	3.0606	0.0022	Unidirectional causality
EPU ≠ > EU	0.7383	− 0.7025	0.4824	
GDP ≠ > EPU	2.0044	1.5492	0.1213	No causality
EPU ≠ > GDP	1.1041	− 0.0518	0.9587	
GDP ≠ > EU	2.5499	2.5193	0.0118	Unidirectional causality
EU ≠ > GDP	1.7452	1.0882	0.2765	
GDPsq ≠ > EU	2.2862	2.0503	0.0403	Bidirectional causality
EU ≠ > GDPsq	9.2020	14.349	0.0000	
TOA ≠ > GDP	3.0911	3.4818	0.0005	Unidirectional causality
GDP ≠ > TOA	1.5157	0.6800	0.4965	
TOA ≠ > EU	3.3774	3.9910	7.E-05	No causality
EU ≠ > TOA	4.1145	5.3019	1.E-07	
TOA ≠ > EPU	1.9128	1.3862	0.1657	No causality
EPU ≠ > TOA	0.6271	− 0.9002	0.3680	
TOA ≠ > EFP	3.5351	4.2715	2.E-05	No causality
EFP ≠ > TOA	1.6556	0.9289	0.3529	

Source: authors’ computations

## Conclusion

This study differs from previous studies by the inclusion of economic policy uncertainty into the tourism-energy-emission nexus and environmental sustainability for ten top-earning countries in international tourism (Germany, Australia, France, Japan, Hong Kong, Italy, the UK, Thailand, Spain, and the USA) over the period 1995–2015. The FMOLS and FDOLS estimation techniques were used to establish the long-run relationship between energy consumption, tourists’ arrivals, economic policy uncertainties, and ecological footprint. Also, the Dumitrescu and Hurlin causality tests were used to find the direction of causality between the study variables. The findings of the study show the EKC does not hold in the ten countries under focus due to the high level of economic development attained by the study countries even as economic literature exempts such countries from the EKC. On the other hand, energy consumption if found to be a driver of environmental degradation and GDP growth does not contribute to environmental degradation especially with the use of non-renewable energy. Likewise, EPU is found to contribute to worsening the environment as well. On the other hand, when the influence of EPU on energy use is recognised, we find that there is an improvement on the environmental quality, which is very interesting. The study also found the absence of causality between EFP and EPU, GDP and EPU, TOA and GDP, EU, EPU, and TOA and EFP. However, the test showed that TOA has an indirect impact on the environment through GDP.

A few policy implications are drawn from the findings of this study. Firstly, the study establishes that energy (non-renewable energy) use worsens the quality of the environment. However, when the influence of policy uncertainty is exerted over energy usage, we find that the environment in the ten countries improves. This further proves the willingness of investors to use renewable energy sources which could improve the environment. Based on these findings, the government of the study countries or policymakers should formulate policies that will discourage or control the consumption of non-renewable energy resources (one such policy is the imposition of carbon tax) and also promote the use of environmentally friendly energy. Such policies will put the study countries on a path to attaining the Sustainable Development Goal 12 by 2030.

Furthermore, the study demonstrates that green growth can be attained as findings showed that economic growth in the ten countries goes alongside improving environmental quality. This demonstrates the commitment of the top ten earning countries to pursue economic growth without depleting the resources in the environment. It, therefore, means that with the proper implementation of environmental protection policies, economic activities can be made to adopt the use of renewable sources for their energy demands and also operate outside activities leading to the destruction of land and the natural habitat, thus improving the ecological footprints.

This study is limited in that it was carried out for a group of countries, as such the findings may not be suitable for policy use in the case of an individual country. It is advised that future studies can be carried out for individual countries. Similarly, future studies could make use of more recent data (covering the years 2016 and above) as they were not available for the study countries at the time this study was carried out.

## Footnotes

1 Source: World Tourism Organization data files (Index Mundi 2018)

2 Real GDP; international tourist arrivals; and energy use are sourced from the World Bank Development Indicator of the World Bank database (https://data.worldbank.org/). WUI = This tab contains the beta version of the historical World Uncertainty Index (WUI) for 82 countries from 1952Q1 to 2019Q3. The tab contains a moving average index. The 3-quarter weighted moving average is computed as follows: 1996Q4 = (1996Q4*0.6) + (1996Q3*0.3) + (1996Q2*0.1)/3.
